# Versatile Functional Energy Metabolism Platform Working From Research to Patient: An Integrated View of Cell Bioenergetics

**DOI:** 10.3389/ftox.2021.750431

**Published:** 2022-02-03

**Authors:** Sylvain Loric, Marc Conti

**Affiliations:** INSERM, Cancer Biology and Therapeutics, CRSA, Saint Antoine University Hospital, Paris, France

**Keywords:** functional metabolism, cell energetics, oxphos, oxidative stress, automatisation

## Abstract

Mitochondrial dysfunctions that were not discovered during preclinical and clinical testing have been responsible for at least restriction of use as far as withdrawal of many drugs. To solve mitochondrial machinery complexity, integrative methodologies combining different data, coupled or not to mathematic modelling into systems biology, could represent a strategic way but are still very hard to implement. These technologies should be accurate and precise to avoid accumulation of errors that can lead to misinterpretations, and then alter prediction efficiency. To address such issue, we have developed a versatile functional energy metabolism platform that can measure quantitatively, in parallel, with a very high precision and accuracy, a high number of biological parameters like substrates or enzyme cascade activities in essential metabolism units (glycolysis, respiratory chain ATP production, oxidative stress...) Its versatility (our platform works on either cell lines or small animals and human samples) allows cell metabolism pathways fine tuning comparison from preclinical to clinical studies. Applied here to OXPHOS and/or oxidative stress as an example, it allows discriminating compounds with acute toxic effects but, most importantly, those inducing low noise chronic ones.

## Introduction

During the last decade, in the field of pharmaco-toxicology, early discovery impairments of adverse effects such as mitochondrial dysfunctions have generated late drug attrition (Nadanaciva and Will, 2011a). As understanding mitochondrial behaviour remains extremely complex and because mitochondrial insults can occur through still unknown mechanisms, it is not surprising that mitochondrial metabolism has often been found to be a target of drug induced toxicity (Nadanaciva and Will, 2011b). Mitochondria produce most of the ATP used by mammalian cells through oxidative phosphorylation (OXPHOS). OXPHOS represents a functional unit located in the inner mitochondrial membrane combining electron transport chain (ETC) through its four complexes (I-IV) and ATP synthesis (Hüttemann et al., 2007). TCA cycle NADH and FADH2 electrons are accepted by ETC complex I or II then transferred to complexes III and IV and later to oxygen. This electron transfer along ETC is coupled with proton transport across mitochondrial inner membrane, establishing the electrochemical gradient that allows ATP generation through complex V (Sherratt, 1991). Mitochondria continuously function to metabolize oxygen and generate superoxide radical and subsequently other reactive oxygen species (ROS) mostly at complex I and III (Chance et al., 1979; Richter et al., 1988; Murphy, 2009). It has been measured that 1–4% of oxygen reacting with ETC is incompletely reduced to ROS (Barja, 1999). This ROS overproduction damages mitochondrial proteins, alters mitochondrial membrane permeability, disrupts mitochondrial calcium homeostasis, induces mitochondrial DNA mutations, leading to interruption of mitochondrial essential function such as ATP production (James and Murphy, 2002; Endlicher et al., 2009). Thus, global free radical production level is likely to guide mitochondria metabolic efficiency (Kramer and Nowak, 1988; Krähenbühl et al., 1994). These ROS deleterious effects are normally counterbalanced by efficient mitochondrial antioxidant defence driven by either enzymatic or non-enzymatic mechanisms (Krähenbühl et al., 1996). When ROS/antioxidant balance is disrupted, every cellular biological system will suffer oxidative stress (OS) that overwhelm mitochondria and damage other cellular components (lipids, proteins, DNA...) thus generating global functional impairment leading to cellular dysfunction then cytotoxicity (Dröge, 2002). This intricate metabolism between ROS generation and ETC function in mitochondria explains why most of mitochondrial dysfunctions (a common term that includes alteration of different metabolic pathways and damage to mitochondrial components) and impaired bioenergetics are strongly implicated in the aging process (Finkel et al., 2000; Short et al., 2005) and the pathogenesis of many chronic illnesses [from metabolic diseases (Bhatti et al., 2017) to various disorders including cancer (Yin et al., 2012; Iommarini et al., 2017), insulin resistance (Houstis et al., 2006; Rocha et al., 2013) and diabetes (Parish and Petersen, 2005; Blake and Trounce, 2014), atherosclerosis (Madamanchi and Runge, 2007), cardiac (Ide et al., 1999; Le Chen and Knowlton, 2011; Ong et al., 2013) and neurodegenerative illnesses (Reddy, 2006; Reddy, 2008; Itoh et al., 2013; Misrani et al., 2021)]. In most cases, there are only subtle metabolic or enzymatic variations that will be indicative of mitochondrial dysfunction. Therefore, it makes sense to combine sensitive ETC analysis with OS evaluation to get an overview of mitochondrial function especially in mitochondrial-dependent diseases.

**FIGURE 1:**
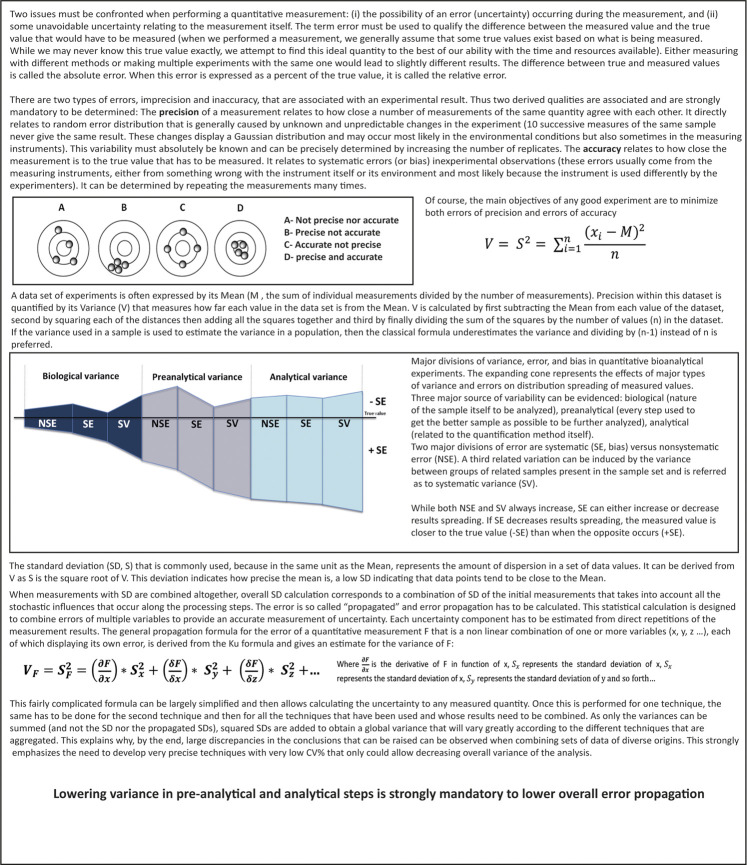
Error definitions and propagation.

**FIGURE 2:**
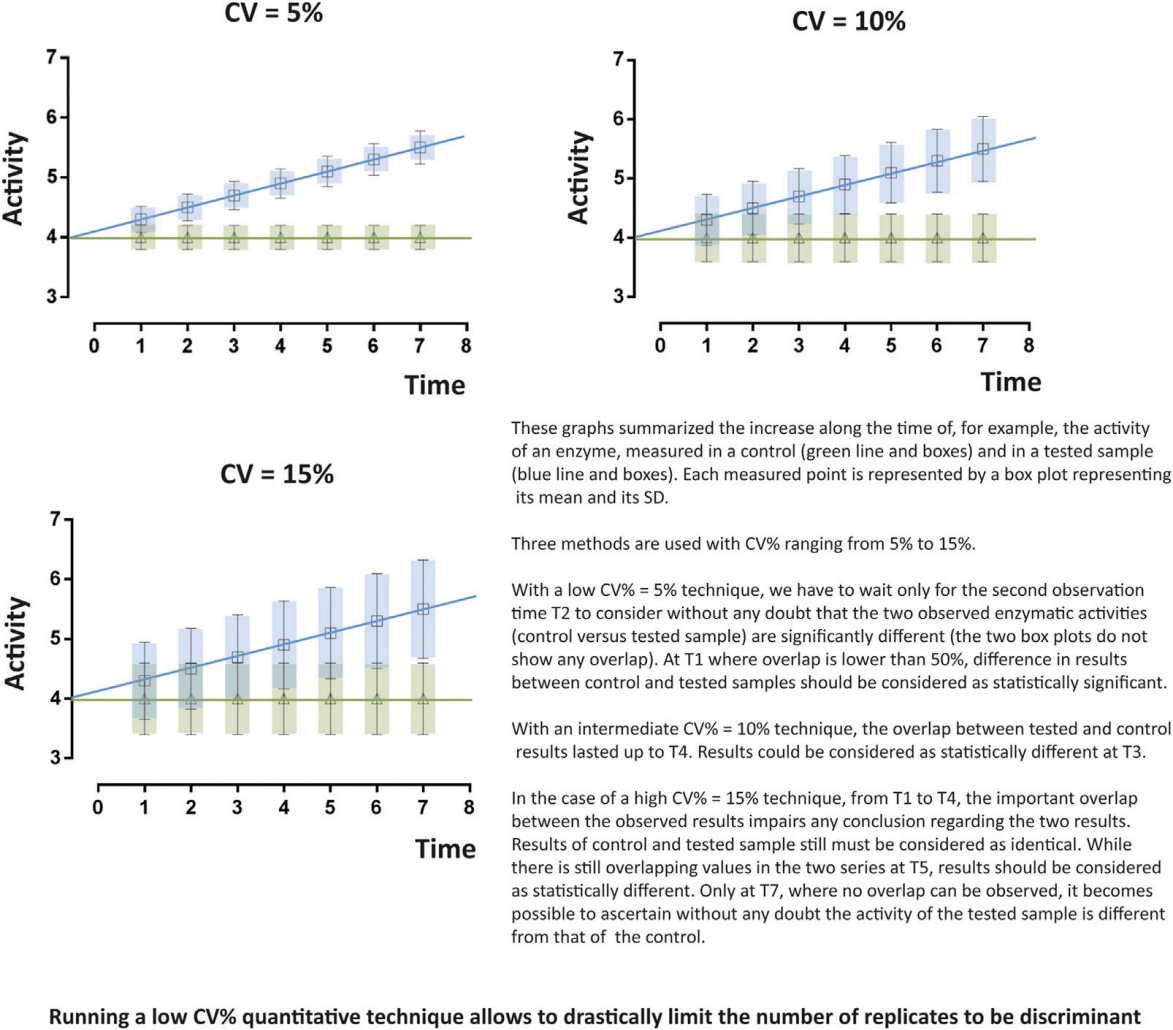
Importance and influence of the coefficient of variation (CV%). The dimensionless and unitless coefficient of variation CV% is often preferred to standard deviation (S) as it is more convenient to describe data spreading regarding a measurement. Indeed, CV%s values are interesting ones as they can be used easily to compare variations of two or more quantitative datasets. Low CV%s, as compared to high ones, allows detecting significant tiny variations. $\mathrm{CV}\%=\frac{S}{M}*100$

To address such intricate issues, only an integrative approach combining different data could represent a strategic way to solve mitochondrial machinery complexity. Such integrative methods are very hard to develop. The technological development of large-scale analysis of genes (genomics), transcripts (transcriptomics), proteins (proteomics) and more recently small metabolites (metabolomics), all referred to the neologism “omics” has been thought to be an interesting tool to understand complex cellular mechanisms and thus find out innovative and active drugs. By generating huge files of data, these techniques were supposed to provide sufficient information to break the gap between genotype and phenotype (Karczewski and Snyder, 2018). Thus, it would be possible to get a holistic overview of cells global operating and even individual networks. For that purpose, statistics and bioinformatics have developed a number of novel methodologies to efficiently process, analyse, integrate and interpret “omics” data (Pesce et al., 2013; Fondi and Liò, 2015; Van Assche et al., 2015; Zapalska-Sozoniuk et al., 2019) that could drive the discovery of new important therapeutic targets that direct development of specific and efficient compounds addressing them (Iskar et al., 2012; Leung et al., 2013). Aside Pharma industry, they were supposed to be massively used in clinical laboratories to discover and then apply new useful biomarkers in either pathologies diagnosis or drug management as companion tests (Strunz et al., 2016). However, “omics” usefulness, either used alone or combined with system biology, remains controversial (Ein-Dor et al., 2006; Lay et al., 2006a; Lay et al., 2006b; Ghosh and Poisson, 2009; Ioannidis, 2010; Sung et al., 2012) and are still limited to research only and not yet used for patient’s health management. A few “omics” protocols have been applied to ETC/OS explorations but are still unable to be used in clinical handling of mitochondrial diseases (Go et al., 2018; Rahman and Rahman, 2018). Indeed, to reach clinical biology criteria, two major points should be considered:

- The first one concerns quantification as an important issue. Whereas random biological errors could not be circumvented, introducing errors at the beginning of the process and accumulating such errors at each step of aggregating data will unavoidably distort mathematic model calculations (Figure 1). As both accuracy and precision of multi-step biological measurement procedures are always the sum of many different elementary errors resulting from either systematic or stochastic influences, integration of results suffering error accumulation would possibly generate false predictions. In a simple comparison system where large difference between two studied cases is expected, even if systematic and stochastic errors do occur, it will be still possible to draw up true concluding remarks. In a complex system in which differences can range within a few percent, no clear answer or no answer at all would be made. This adverse error propagation can be approximated by statistical calculations but unfortunately is rarely performed (Figure 1) (Moseley, 2013; Noack and Wiechert, 2014). If these results are sustained by economic considerations, like in the field of drug discovery, or by vital ones, in the medical field, availability of accurate quantitative methods looks like a mandatory challenge.
- The second one concerns measurement reproducibility (Perng and Aslibekyan, 2020). A survey published approximately two years ago reported that more than 70% of researchers have tried and failed to reproduce another scientist’s measurement (Baker, 2016). This is quite impossible in clinical biology as reproducibility of results is mandatory and mainly the consequence of low CV% (either intra- or inter-assays CV%) of automated devices that display sufficient power to significantly detect tiny variations (Figure 2). In contrast, high CVs have low power to detect small-scale differences and, the only way to increase power with high CVs techniques relies in increasing the number of replicates. Using tests with the least inherent variability will induce the least replication and thus will be the most cost-effective (for example, in every clinical biochemistry laboratory in the world, blood measurement of every parameter is only performed once as test CV% are very low). Developing the lowest CV% techniques in research or in drug development strategies will increase confidence in data. Study results will then be considered with the most attention and avoid criticisms.

We report here, as a significant example, the development of a robust and accurate system to evaluate in a combined fashion either mitochondrial function or subsequent OS in different situations (cell culture, tissues, blood samples). This system, based on the use of a multiparametric analyzer (normally devoted to clinical biochemistry laboratories) allows to measure a panel of several ETC (complexes I to V) and OS (SOD1,2, GPX, Catalase, Glutathione reductase) markers on a unique sample. Every test is performed in parallel with high precision, without any bias due to either inter-experiments variability or sample degradation. This measurement method not only allows to precisely evaluate in parallel on number of samples both mitochondrial function and OS involvement in an integrative way, but it also allows comparing drug effects in a dynamic fashion. Because of its versatility, it can be used not only in heath management of patients but also in research on cell culture and animal testing.

## Material and Methods

### Reagents

All chemicals purchased from Sigma. RPMI-1640 media were purchased from Gibco/ThermoFischer. Penicillin-Streptomycin solution (100x) and 1x PBS were purchased from Corning. Standard cell media was made by adding 10% serum (either FCS or DC-FCS) to 500 ml RPMI-1640, together with pen/strep and L-glutamine (ThermoFischer).

### Cell Culture and Harvest

VCaP and PC3 prostate cancer (CaP) cell lines were purchased from ATCC, PNT1A from Sigma and were routinely maintained in RPMI medium containing 1 g/L glucose and supplemented with 10% fetal calf serum. PNT1A is an SV40 immortalized cell line, derived from normal prostate, mimicking normal epithelial prostate cells. VCaP cell line has been derived from CaP patient lumbar vertebral metastases, and behaves as a classical androgen-sensitive prostate adenocarcinoma cell line. PC3 cell line derived from bone metastasis of grade IV CaP patient, and is no more responsive to androgens, glucocorticoids or fibroblast growth factors.

Cells were scraped using rubber policeman then washed two times with cold PBS (Trypsin was avoided as it generates cellular stress). Cell suspension was then centrifuged at 400 *g* for 5 min. After careful supernatant withdrawing, cell pellet was snap frozen in liquid nitrogen and kept at −80°C. Before treatment, the resulting pellet was resuspended in 20 mmol/L phosphate buffer (pH 7.4) then homogenized on ice in a Bandelin Sonoplus ultrasonic homogenizer for 20 s at 30% power. An aliquot was immediately collected on 2% metaphosphoric acid for further ATP and GSH measurements. This acidic suspension was centrifuged at 13,000 rpm for 15 min at 4°C and supernatants were collected, aliquoted, and stored at −80°C until analysis. The remaining preparation was centrifuged a final time at 600 *g* for 10 min, and the supernatants were kept at 4°C until analysis on the automated system.

For 5 g/L time dependent experiments, cells were initially grown in 1 g/L. Then after washing them two times in PBS, 5 g/L medium was added for 10 min (t10 m), t6 h and t12 h. At the end of the period, cells were washed again in cold PBS as described.

### Tissue Sample Preparation

Tissue biopsies of gastrocnemius muscle of Sprague Dawley rats (Charles River) have been originally obtained for another study. Rat housing, surgical procedures, and assessment of analgesia have been performed in strict agreement with the regulations set out in EU laws and approved by local university ethical and animal care committees. Efforts were made to minimize animal suffering and to only use the number of animals necessary to produce reliable scientific data. Animals were housed in smooth-bottomed plastic cages at 22°C with a 12 h light/dark cycle. Food and water were available ad libitum. At the end of the study, rats were anesthetized with isoflurane anesthesia and sacrificed by exsanguination [complete blood draw (about 1 ml) from the vena cava, followed by perfusion with phosphate buffered saline]. Gastrocnemius samples were obtained as follows. A 2 cm incision was performed over the gastrocnemius muscle and 0.5 cm^3^ segments of muscle tissue were obtained under sterile conditions. Muscle tissue samples were immediately snap frozen and stored at −70°C.

Tissue samples from *Caenorhabditis elegans* were obtained as follows. The *C. elegans* strain N2 was obtained from *Caenorhabditis* Genetics Center. Worms were cultured on bacterial lawns containing OP50 bacteria on NGM plates at 20°C according to standard methods (Nass and Hamza, 2007). For rotenone experiments, NGM plates were pre-coated with rotenone in DMSO to achieve a concentration of 2 or 4 μM, a concentration that did not cause any mortality throughout the course of experiments. After synchronization, a cohort of 1st larval stage (L1) was obtained. After washing, L1 worms were then loaded on rotenone-coated plates. After 24 h, worms were separated, snap frozen then stored at −70°C.

For our retrospective analysis, frozen tissue samples were weighed prior homogenization using an Ultra-turrax Ika T25 for 30 s in an adequate volume of 20 mM phosphate buffer saline pH7.4. An aliquot of the suspended material was immediately collected on 2% metaphosphoric acid for further ATP and GSH measurements. Samples were then centrifuged at 600 g for 10 min at 4°C, and the supernatants were kept on wet ice until analysis on the automated system.

### White Blood Cell Preparation

Antecubital human blood draws were performed in one 5 ml EDTA vacutainer. A volume of blood was then diluted with the same volume of NaCl 0.9%, (V/V). One volume of this diluted blood was carefully poured onto one volume of Lymphoprep (Biovision). After centrifugation at 4,000 rpm 20 min at 20°C, plasma was discarded, and the lymphocyte layer was transferred for washing with NaCl 0.9%. After shaking and centrifugation at 600 g for 5 min at + 4°C, supernatant is removed, and cell pellet was frozen as quickly as possible at −80°C.

Before analysis, the resulting pellet was resuspended in 20 mmol/L phosphate buffer (pH 7.4) then homogenized on ice in a Bandelin Sonoplus ultrasonic homogenizer for 20 s at 30% power. An aliquot was immediately collected on 2% metaphosphoric acid for further ATP and GSH measurements. This acidic suspension was centrifuged at 13,000 rpm for 15 min at 4°C and supernatants were collected, aliquoted, and stored at −80°C until analysis. The remaining preparation was centrifuged a final time at 600 *g* for 10 min, and the supernatants were kept at 4°C until analysis on the automated apparatus.

Three blood samples were analyzed. The first one (referred as normal one) was obtained from a normal volunteer devoided of any known illnesses (male, aged 23). Two young patients were also analyzed: One is a 2.5 year-old male patient who was screened for potential Leigh syndrome (LS) at the age of 7 months presenting muscle hypotonia, ataxia and developmental delay (and already related to MTND5 mutation). The second one is a 14 year-old male suffering seizures and myasthenia who was screened for potential late-onset LS syndrome.

### Automate Apparatus

The apparatus, a Roche/Hitachi-ModularP analyzer, normally dedicated to routine clinical biochemistry, consists in an automated spectrophotometer that can analyze up to 300 samples, with continuous loading of five-position sample racks and a maximum capacity of 800 tests/h. It can perform in parallel a maximum of 86 different photometric tests. Each programmed assay can use up to 4 different reagents. First reagents are dispensed into the cuvette as few as 10 s after the sample. Other reagents can be dispensed between 1 and 10 min, after the first one. Absorbance readings are taken every 20 s at a specified wavelength in a kinetic fashion, allowing very precise reaction follow-up. Automation allows launching one assay every 6 s. Thus, a multi-panel of analysis consisting of more than 20 assays may be launched in 2 min time limiting the biological sample evolution. All measurements are performed on a same sample at the same time and as all results are given in less than 30 min, it becomes possible with the same apparatus and reagents to rerun samples, whose results seem unfitting, without time degradation between series.

These technical specificities give these biochemical assays a very high reliability and generate small CV% variation coefficients. The lower the CV, the less variation there is and the higher the confidence in the results given by this precise test, so there is no need to iterate the test to ascertain the validity of results, saving sample volumes and being able to carry out all the experiments on the same aliquot. With such accuracy, tests panel can be performed on as few as 3 10^6^ cells or 5–10 mg biopsies. Last, it is possible to measure in the same series either cell homogenates, biopsies or tissues homogenates from different species, nucleated cells and red blood cells hemolysates (one 5 ml vacutainer is sufficient).

### Methods

Originally, all the techniques reported below have been developed and used manually for years, either for patients in the context of metabolic exploration of innate pathologies in our hospital clinical biology laboratory, or for our research work. As manual techniques are largely time consuming, need large samples volumes and do not preserve material stability, we were prompted to transfer these techniques to automated analyzers. To adapt them, we had to modify volume ratios of samples and reagent concentrations because of the stringent mechanical constraints of the Roche apparatus. Total reaction volume is strictly limited to 250 µL. Sample volume cannot exceed 20 µL. Only two reagents can be used to perform the analysis. The volume of the first reagent, generally the one that starts the reaction and measures non-specific reactions, cannot exceed 190 µL. The second one, that brings specificity, is limited to 50 µL. Therefore, to use 20 µL of test sample, it was therefore necessary to reduce the number of reagents to only two by making premixes whose stability need to be tested prior any experiment. Then, the concentrations of the different reagent components have to be tightly adapted so that the final concentrations in the reaction medium remained identical to those of the reagents used manually. Of course, each step of this process was carefully compared to reference manual technique results then validated. This process took place during our 30 years of experiences in the field. Quality controls (QC) (both frozen mix of crude enzymes and tissue homogenates) were performed during each run of analysis and Levey-Jennings graphs were used to monitor QC values.

### Oxidative Phosphorylation Complexes Measurements

**FIGURE 3:**
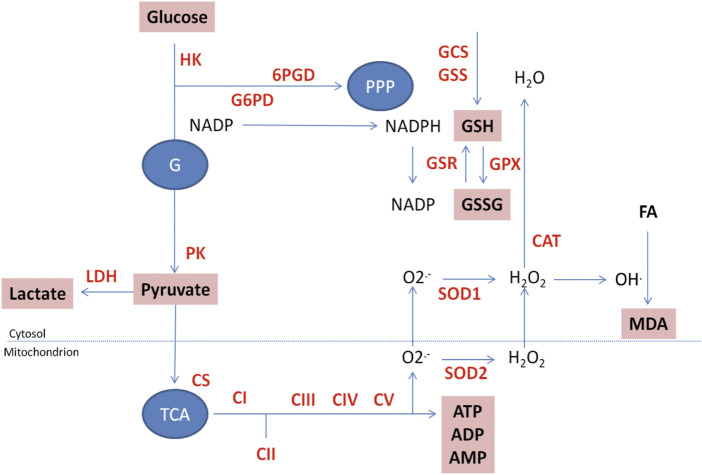
Functional energy metabolism: visualization of metabolic cascades. To date, 22 analytes and enzymes, presented on the schema above, are measured in parallel on the platform. GSH and GSSG, ATP, ADP, and AMP are measured by capillary electrophoresis. All CV% of the different techniques are below 5%. HK: hexokinase; G6PD: glucose 6-P dehydrogenase; 6PGD: 6P-gluconolactone dehydrogenase; PK: pyruvate kinase; LDH: lactate dehydrogenase; CS: citrate synthase; CI to CV: ETC complexes I to V; SOD1: CuZn superoxide dismutase; SOD2: MnSOD; CAT: catalase; GSR: glutathione disulfide reductase; GPX: glutathione peroxidase; GCS: *γ*-glutamyl cysteinyl synthase; GSS: glutathione synthetase; PK: Pyruvate kinase; LDH: lactate dehydrogenase. GSH: reduced glutathione; GSSG: oxidized glutathione; MDA: malonedialdehyde. G: glycolysis; PPP: pentose phosphate pathway, TCA: tricarboxylic acid cycle; FA: Fatty acids. Measured enzymes in red; measured molecules in bold in boxes.

ETC complexes, coenzyme Q, and cytochrome C transform energy transduction from respiratory substrates to a proton motive gradient needed for ATP synthesis (Figure 3). This pathway is made up of five multi-enzymatic complexes (complexes I–V) whose function is essential for ATP production (complex V). All respiratory chain complex assays were based on methods described by Kramer (Kramer and Nowak, 1988) and Krakenbuhl (Krähenbühl et al., 1994; Krähenbühl et al., 1996), both modified to match our apparatus requirements. The activities of all the complexes in each sample were normalized by the amount of protein or referred to citrate synthase activity to allow sample comparison.

• Complex I activity measurement (NADH dehydrogenase)

Briefly, reduced nicotinamide adenine dinucleotide phosphate (NADH)-ubiquinone reductase activity (complex I) was measured by following the disappearance of NADH using rotenone as a specific inhibitor to ensure the specificity of the assay.

• Complex II activity measurement (succinate dehydrogenase)

Complex II activity, succinate-ubiquinone reductase, was assayed through the reduction of 2,6-dichlorophenolindophenol, a final electron acceptor, after the addition of succinate.

• Complex III activity measurement (ubiquinone bc1 complex)

The activity of complex III, ubiquinone-cytochrome c reductase, was determined by assaying the rate of reduction of cytochrome c.

• Complex IV activity measurement (Cytochrome C oxidase)

The cytochrome c oxidase (complex IV) activity was based on the same assay as for complex III using potassium cyanide to inhibit the activity of this enzyme.

• Complex V activity measurement (ATP synthase)

Complex V activity was measured according to a method coupling ADP production to NADH disappearance through the conversion of phosphoenol-pyruvate into pyruvate then into lactate (Rustin et al., 1993).

• Citrate synthase (CS) activity measurement

The activity of CS was assayed as described previously (Itoh and Srere, 1970) with the reduction of DTNB caused by the de-acetylation of acetyl-CoA.

• Evaluation of the energetic capability

ATP/ADP/AMP cellular levels were assessed by capillary electrophoresis after protein precipitation with 2% PCA (Uhrová et al., 1996; Markuszewski et al., 2003). Two calculations can then be derived:

- Adenylate energy charge (AEC) is a measure of disposable energy at a given moment in the cell. AEC = [(ATP)+1/2(ADP)]/[(ATP)+(ADP)+(AMP)].
- Total adenine nucleotides (TAN) evaluation gives an idea of the pool of adenine nucleotide available in the cell. TAN = AMP + ADP + ATP
• Assessment of anaerobic glycolysis

When mitochondria cannot supply sufficient ATP for cell metabolism, anaerobic glycolysis is activated. We therefore see in the activation of anaerobic glycolysis an indirect proof of mitochondrial dysfunction. G6PD catalyzes the production of ATP in anaerobic conditions using glucose as substrate and generating lactate. It was assayed as described by Beutler (Beutler and Mitchell, 1968).

### OS Pathway Measurements

To study the reduction of O_2_ into water, we measured the activities of each of the main antioxidant enzymes and antioxidant compounds in this pathway (Figure 3). When the superoxide anion is produced, this highly toxic entity must be quickly detoxified. Superoxide dismutases (SOD) will reduce it into hydrogen peroxide (H_2_O_2_), an even more oxidizing entity than superoxide anion itself that will be even more toxic. Thus, it is mandatory to combine SOD measurement with the quantification of the activities of the two enzymes [glutathione peroxidase (GPX) and catalase (CAT)] able to reduce H_2_O_2_ into a non-toxic molecule, namely water. As GPX need glutathione, oxidized (GSSG) and reduced glutathione (GSH) must be quantified to evaluate if GSH is in sufficient amount to allow H_2_O_2_ reduction. As GSH is either produced through glutamate-cysteine ligase (GCS) and glutathione synthetase (GSS) synthesis pathway or reduced from GSSG by NADPH-dependent glutathione reductase (GSR), these three enzymes must be quantified. In addition, as NADPH is mandatory for glutathione reduction, Glucose-6-Phosphate Dehydrogenase (G6PD) activity is required. Last, to evaluate if antioxidant defense is efficient, and because lipid peroxidation is the OS primary target, the measurement of a specific marker like malonedialdehyde (MDA) is required.

• Assessment of superoxide radical reduction

To study O2 reduction to H_2_O_2_, cytosolic SOD (Cu/Zn-SOD, SOD1) and mitochondrial SOD (Mn-SOD, SOD2) activities were both measured. Using a slightly modified protocol based on the method developed by McCord and Fridovich (McCord and Fridovich, 1969; Huet et al., 2011), total SOD was measured at pH7.8 and SOD1 was measured at pH10.2. SOD2 activity was calculated as the difference between total SOD and SOD1 activities.

• Assessment of H_2_O_2_ reduction

H_2_O_2_ is reduced to water mainly by the glutathione antioxidant pathway. GPX activity was measured using a method based on the one developed by Paglia and Valentine (Paglia and Valentine, 1967). CAT activation being another mode of H_2_O_2_ removal, CAT activity was measured using derived method from the one developed by Johansson (Johansson and Håkan Borg, 1988).

• Assessment of the Glutathione antioxidant pathway

This pathway includes the antioxidant enzymes GPX, GSR, G6PD, and non-enzymatic antioxidant compounds such as reduced and oxidized glutathione (GSH and GSSG).

GSR and G6PD activities were estimated as already described by Beutler (Beutler and Mitchell, 1968). Glutathione synthesis was estimated by measuring the activity of GCS and GSS as described previously (Huang et al., 1993).

GSH and GSSG concentrations were assayed by capillary zone electrophoresis after protein precipitation with 2% PCA (Muscari et al., 1998; Serru et al., 2001). Total glutathione is calculated as the sum of GSH plus GSSG.

• Lipid peroxidation assay

MDA concentration was measured by spectrofluorometry as previously described (Conti et al., 1991). Briefly, the sample was treated with diethylthiobarbituric acid (DETBA) and the fluorescent compound was then extracted with butanol and determined by synchronous fluorescence spectroscopy. MDA in each sample was also normalized with total proteins assayed by the method of Bradford (Bradford, 1976).

### Other Measurements

• Lactate dehydrogenase (LDH) activity measurement

The activity of LDH was determined using the Cobas commercial LDHI2 kit (Roche). This spectrophotometric method, based on the rate of the NADH formation that is directly proportional to the catalytic LDH activity, is derived from the formulation recommended by the IFCC (Schumann et al., 2002) and was optimized for performance and stability on Roche apparatus.

• Pyruvate kinase (PK) activity measurement

Pyruvate kinase activity was assayed spectrophotometrically using a derived method measuring the decrease in absorbance at 340 nm using a coupled system with LDH and NADH as described previously (Carbonell et al., 1973). After stabilization between homogenates and reagents, phosphoenolpyruvate is used as the starting reagent.

• Lactate measurement

Lactate concentration was measured using the Cobas commercial LACT2 kit. Briefly, L-lactate is oxidized to pyruvate by the specific enzyme lactate oxidase (LOD). Peroxidase (POD) is used to generate a colored dye using the hydrogen peroxide generated in the first reaction (Trinder, 1969).

As mentioned in Figure 2, low CV% are mandatory to compare enzymatic activities. Analysis of the intra-assay variability of the ETC/OS panel tests was performed (the same cell extract was measured 20 times in the same experiment). For the 22 parameters, intra-assay CV% vary from 2,7 to 5,5% (data not shown). Inter-assay variability (the same cell extract was frozen, kept at −80°C and was thawed then analyzed 10 more times in 10 different experiments) of all the tests of the ETC/OS panel varies from 3,5 to 7,7%, which is quite acceptable for inter-assay measurements (data not shown). Once validated, tests can be used to characterize cell lines.

### Data Analysis

All experiment data were processed by GraphPad Prism 8.3 software. The results were shown as median ± SD or SEM. Non-parametric tests were used in all different groups, and *p* < 0.05 was considered as statistically significant (Snedecor and Cochran, 1967).

## Results

Combined analysis of ETC and OS supposed to quantify the different elements represented in Figure 3. Additionally, to ETC and OS pathways, a glance at glycolysis is made through PK, LDH, and lactate measurements. Except GSH/GSSG, ATP, and MDA, every analysis has been performed on the same sample and in parallel on the Roche automated apparatus.

### Cell (or Tissue) Metabolism Comparison

**FIGURE 4:**
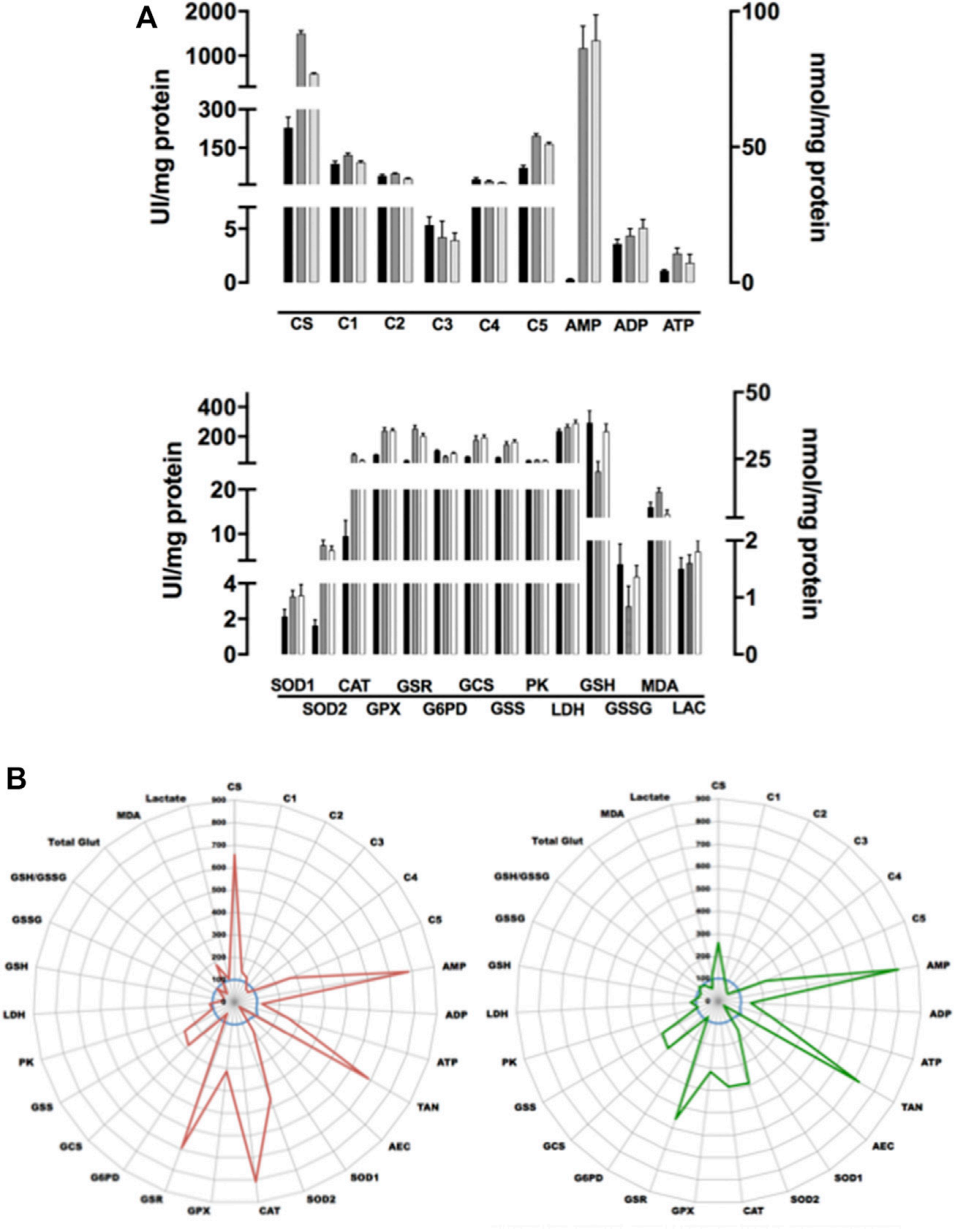
Comparative analysis of three prostate cell lines. **(A)**: Figure 4A depicts results of the three cell lines (mean of five different experiments) in conventional box plots showing mean and standard deviation. PNT1A cells are in black bars, VCaP in grey and PC3 in white ones. Upper figure: AMP, ADP, and ATP are plot to right axis while all other parameters refer to left one. Lower figure: GSH, GSSG, MDA, and LAC are plot to right axis while all other parameters refer to left one. **(B)**: Radar graph depicting the metabolic results of the three cell lines (prostate PNT1A, blue line, normal control as 100%; VCaP cells, red line and PC3 cells, green line). Each point represents the mean of 5 independent experiments. All enzyme results are expressed in UI/g protein, lactate, glutathione and MDA respectively in mmol, μmol, nmol/g protein. The scale on the radar graph represents % of variations versus control. TAN: total adenylate (ATP + ADP + AMP), AEC: adenylate energy charge (ATP+1/2ADP)/TAN.

As an example, and because our research mainly focuses on prostate cancer evolution, ETC/OS panel was firstly performed on three well-known prostate cell lines, namely PNT1A, VCaP and PC3 cells. Figure 4A depicts results of the three cell lines in conventional bar graphs showing standard deviation. While less informative in term of true quantitative results, radar panel is more convenient than bar graphs as it allows immediate comparison of different cultured cells at their metabolism level. Therefore, using that graphic representation, it becomes easy to compare a control cell line (here, the normal prostate PNT1A cells) to its cancer neighbors (VCaP and PC3 cells) (Figure 4B). When normalized with sample protein content, and compared to PNT1A, VCaP and PC3 cells undergo a very significant increase in ETC complex V activity (*p* < 0.001) while other I to IV complex activities remain stable or slightly decreased (CIII and CIV activities in PC3 cells). This increase in ATP synthase activity leads to significant ATP increase in VCaP cells (*p* < 0.001) while ATP is only very slightly increased in PC3 cells (not significant). A very interesting finding is the dramatic AMP increase both VCaP and PC3 cells experienced (*p* < 0.0001) indicating that adenosine metabolism is likely to be crucial in CaP progression. Adenylate kinase that directs adenosine phosphorylation into AMP (the substrate of the central metabolic regulator AMP kinase) has been shown to be important in cancer cells capability to rewire bioenergetics and metabolic signaling circuits to fuel their uncontrolled proliferation and metastasis (Klepinin et al., 2020). As adenylate energy charge (AEC) is decreased in both cells, it is tempting to speculate AMPK is certainly activated in these cells to restore AEC when intracellular levels of ATP drop down (Iommarini et al., 2017). When activated, AMPK promotes catabolic processes and mitochondrial biogenesis. In both VCaP and PC3 cells, it seems that mitogenesis is likely to be increased (more importantly in VCaP than in PC3 cells) as citrate synthase activity, a constitutive enzyme of the mitochondrial matrix and a validated marker of the mitochondrial content (Vigelsø et al., 2014), appears to be increased. Interestingly, these cells do not enter a glycolytic process to restore ATP production as PK and LDH activities remain stable without production of increasing amounts of lactate.

Concerning OS, it is interesting to notice that both VCaP and PC3 undergo an important oxidative stress as all antioxidant enzymes activities are increased (SOD2 and CAT, *p* < 0.0001; SOD1 and GPX, *p* < 0,001 when compared to PNT1A) leading to lipid peroxidation in VCaP cells (MDA concentration increases). Stress seems to be largely mitochondrial as higher SOD2 activity than SOD1 can be found in both cells. In the meantime, GSR reductase activity increases to refill GSH and GSH synthesis is also induced (GCS activity increases).

### Drug Effect on Energy Metabolism

With such test panel, it is also possible to easily compare drug concentrations respective effects. Here, we use glucose as a well-known toxicant for cells at high concentrations. It is recognized that high glucose concentration can generate OS and induce mitochondrial dysfunction (Blake and Trounce, 2014; Liemburg-Apers et al., 2015) and is responsible of well-described oxidation particularly in many organs (kidney, vessels, eyes, heart, brain) (Forbes and Cooper, 2013). Glucose effect on cells remains an interesting issue as a major nutriment, its role in the context of tumor cells is still a matter of debate (Kamarajugadda et al., 2012; Yin et al., 2012).

**FIGURE 5:**
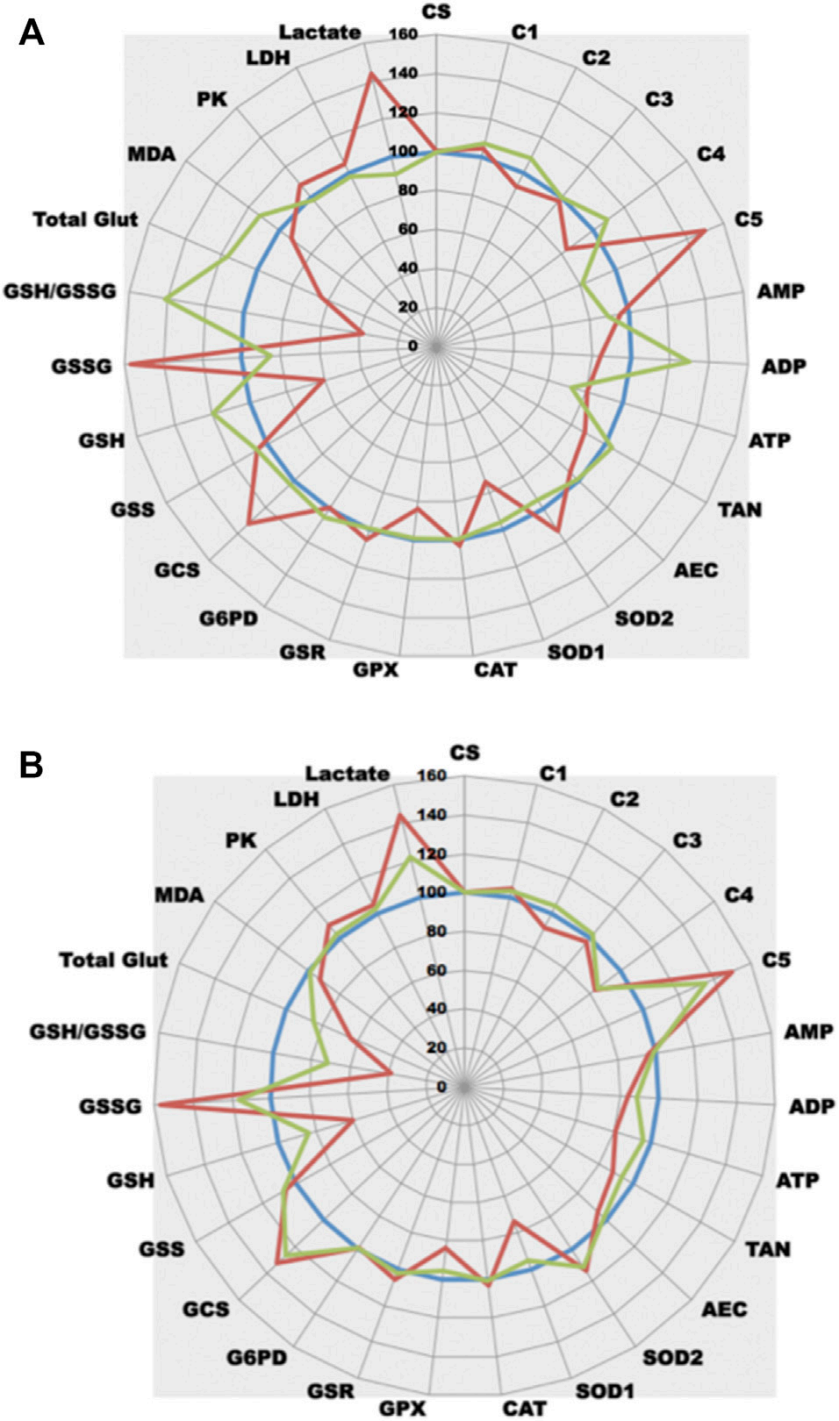
Comparative analysis of the effect of glucose on cell metabolism. **(A)** Three different glucose concentrations were tested at t12h (1 g/L blue line, normal control as 100%; 2.5 g/L green line; 5 g/L red line) in a cell line model (prostate PNT1A cells). Each point represents the mean of 5 independent experiments. **(B)** A unique dose of 5 g/L glucose was tested at 3 different times t10m (blue line, normal control as 100% obtained with 10 min contact of cells with 5 g/L medium), t6h (green line) and t12h (red line) in a cell line model (prostate PNT1A cells). Each point represents the mean of 5 independent experiments.

As shown in Figure 5A, three glucose concentrations varying from 1 g/L (the normal one) to 5 g/L (the classical glucose concentration of the universally used DMEM medium for cell culture) were tested for 12 h on PNT1A cells. The highest glucose concentration decreases ETC (complex II, III, and IV activities) with a drop in ATP synthesis (about 30%) that try to be compensated by a large increase in complex V activity (+ 150%). It is interesting to notice that in cells under 2,5 g/L glucose, ETC remains unchanged when compared to control while ATP is already decreased. TAN in these cells is conserved as ADP concentration largely increases. High glucose generates also an important OS, mainly of mitochondrial origin (while SOD1 activity remained unchanged, an increased in SOD2 was observed). This OS induces an important GSH depletion and a dramatic increase in GSSG at 5 g/L. To efficiently reduce GSSG, GSR reducing activity increases slightly. In parallel, to fill in GSH, GSH production also increases (GCS activity largely increases). It is interesting to notice that up to 2,5 g/L, OS can be controlled by antioxidant defenses (while GSH refilling is already in process, GSH is still in sufficient amount). Additionally, as one would expect, large glucose moiety generates through glycolysis important amounts of pyruvate. This neosynthesized pyruvate will be transformed into lactate (lactate concentrations are increased) rather than being used in the TCA cycle.

**FIGURE 6:**
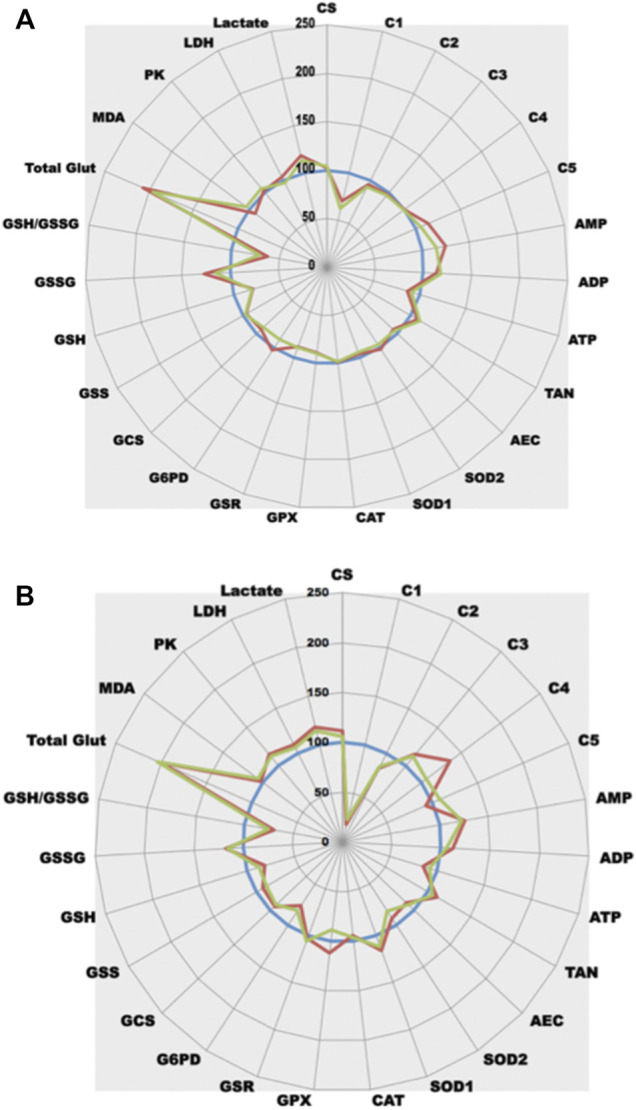
Comparative analysis of the effect of rotenone on cell metabolism. **(A)**-Rotenone-induced complex I dysfunction in PNT1A cells (blue line, control = 100%; green line, + rotenone 5 nM; red line, + rotenone 50 nM). **(B)**-Rotenone effect in an animal model (Caenorhabditis elegans) (blue line, control = 100%; green line, + rotenone 50 nM; red line, + rotenone 100 nM).

Glucose behaves as a global toxicant as it unbalances normal metabolism at different levels (it modulates energy production, but it also generates an important OS). We have also tested rotenone, a well-known specific inhibitor of ETC complex I. As already shown for glucose, the use of a specific drug, like rotenone, not only generates dysfunction of the rotenone target enzymes but also modifies cell metabolism (Figure 6A). As a clear resultant of rotenone action, complex I activity largely drops down. Consequently, ATP production decreases and ATP precursors (ADP, AMP) accumulate leading to an unchanged TAN. It is interesting to observe that one of the side effects of rotenone is an important generation of GSSG, a drop in GSH content and further MDA increase suggesting an associated OS could occur. This OS is likely to be a consequence of the mitochondrial electron leaking as a slight SOD2 increase is also present. A partial GPX inhibition is also observed, contributing to OS settlement. An increase in NADPH synthesis occurs (G6PD activity increases) to unlimit GSR reductive capabilities to replenish the GSH pool that is consumed to counterfight OS. Interestingly, as ETC complex I dysfunctions, pyruvate do not enter mitochondria for TCA then OXPHOS but is transformed by LDH into lactate.

This puzzling effect of rotenone can be evidenced not only on cultured cells but also can be confirmed in animal models as all animals share the same enzymes (with slight modifications of Km and Vmax values as shown in the Brenda database (https://www.brenda-enzymes.org/). The same drop in *Caenorhabditis* Elegans ETC complex I activity can be observed (Figure 6B), contributing to lower ATP synthesis. As for cultured cells, ETC dysfunction also generates OS in animals that seems to be more of cytosolic origin (SOD1 increase rather than SOD2). A same drop in GSH related either to GSH expenditure (GSH synthesis is decreased) or decreased NADPH synthesis (a significant drop in G6PD activity is observed) can be evidenced. *In vivo*, GPX inhibition does not appear. Likewise, an increase in glycolysis (increased PK activity) with endpoint lactate production can be evidenced.

**FIGURE 7:**
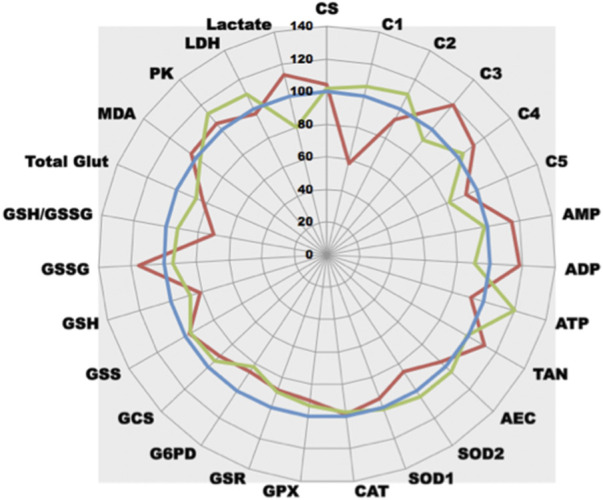
Comparative analysis of the effect of complex I deficiency. After blood draw and further white blood cells careful separation, white blood cells of patients were compared (normal patient as control, blue line, control = 100%; green line, patient 1; red line, patient 2).

After white blood cell isolation, the test panel can be also used to evidence complex I dysfunction in human patients (Figure 7). As compared to a normal control patient, patient 2 displays an important drop in complex I activity leading to a defect in ATP synthesis. Interestingly, he also shows a reactive increase in complex III and IV activities suggesting that ETC electron flux is activated through complex II. As for the rotenone-induced effect, GSH content is also decreased with related GSSG increase, certainly due to ETC dysfunction associated OS. Patient 1 who was addressed for potential complex I dysfunction displays a rather normal pattern. These results on human samples confirm those observed when complex I inhibition occurs either *in vitro* or *in vivo*. Interestingly, Patient 1 undergoes a global increase of ETC activity leading to increasing ATP production. This increased in OXPHOS is associated with an increased glycolysis (PK activity rises). More pyruvate is produced but most of this pyruvate enters mitochondria (lactate concentration is largely decreased).

### Time-Dependent Changes of Energy Metabolism

Static data aside, it is also possible to iterate quantifications and observe metabolic dynamic changes, low CV% allowing to unravel slight modifications. When compared at three different times the treated cell line (Figure 5B), it becomes possible to observe the progressive effect of high glucose concentrations on cell metabolism. Figure 5B depicts time effect of 5 g/L glucose concentrations on ETC/OS metabolisms. ETC complex I and III activities remain stable at t6 h while complexes II and IV are largely decreased at t12 h. Important complex V activation already appears at t6 h (mitochondrial OS seems an early event as at t6 h SOD2 activity is also increased). Interestingly, the observed but unexplained t12 h SOD1 and GPX decrease are already present at t6 h. Glutathione reductase and synthase activities are prematurely induced at t6 h. GSR was slightly increased (about 10%). Interestingly, a 40% increase in GCS occurs to efficiently counterbalance the beginning GSH depletion. Finally, redistribution of pyruvate through LDH pathway seems to be an early event since lactate begins to be largely produced at t6 h.

### Large Scale Analysis of Energy Metabolism

**FIGURE 8:**
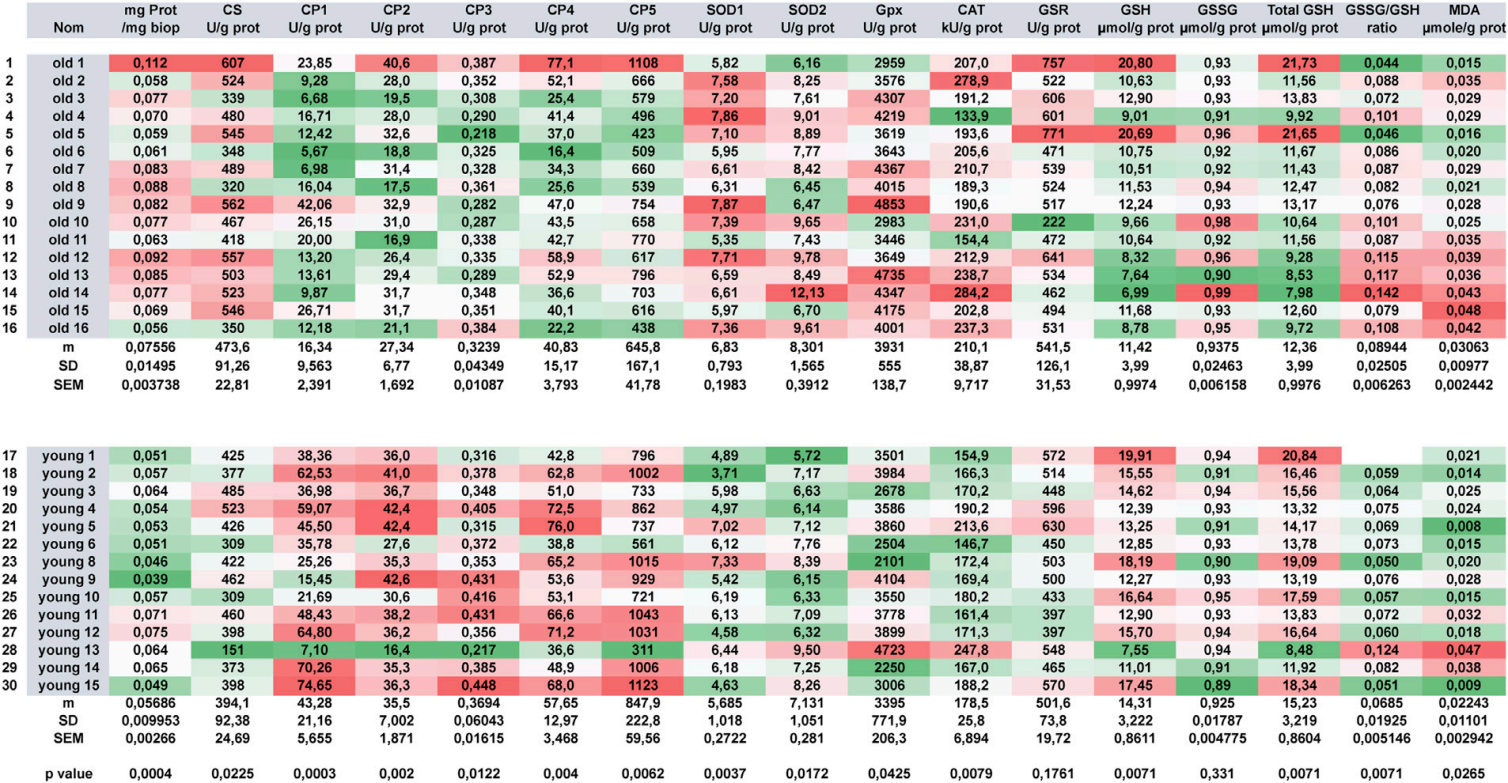
Comparative analysis of functional metabolic activities of rat muscles. Two series of old (n = 16) and young (n = 14) were metabolically analyzed. A red white green 3-color diverging scale heat map (with high values getting the red color and low values getting the green ones) was used to represent the results.

Automation allows the combined analysis of a series of cell metabolism markers. As we used analyzers normally devoted to human sample analysis, their high throughput performance allows us to analyze such large panel in parallel with number of different samples. Figure 8 depicts the results that can be obtained on rat tissues (here, gastrocnemius muscle tissue samples). As all the different muscle extracts are launch altogether on the machine, no batch effect can be observed as all the reagents are identical during the time course of the different tests. As they are kept on the machine at low temperature, neither reagent modification nor degradation can be observed. Two adult rat groups were analyzed: the first one comprised 16 elder rats (mean age 1,76 years) and the second 14 younger adult ones (mean age 0,92 years). Careful analysis of results shows that muscles from old and young rats largely differ from young ones in term of metabolism (except for GSR activity and GSSG content where no significant difference can be evidenced, all other metabolic markers statistically differ between the two groups with *p* values ranging from *p* < 0.0001 (CI) to *p* < 0,05 (CS, SOD2, GPX, MDA). Heat map show that globally elder rat’s muscles display lower ETC and higher OS than younger ones where we observed the exact opposite with high ETC and low corresponding OS. In elder rats, OS induces increasing antioxidant enzymes. SOD1 and SOD2 are both elevated indicating the existence of a mixed cytosolic/mitochondrial OS. GPX and CAT are also increased generating an important GSH depletion. As GSH is not sufficient to counterbalance OS, GSSG increases and peroxidation occurs (except for young rat 13, the highest MDA concentrations are all found in elder rats). As no batch effect can be evidenced in the different series, the heat map allows at a glance to discriminate outliers from the general population: elder rat 1 can be seen immediately as an outlier in his own group as well as younger rat 13 in the youngest one. Unfortunately, no clear explanations could account for these discrepancies. These results are in accordance with what has been already published. Altered mitochondrial function has been linked to aging-related declines in performance (Short et al., 2005) and was more recently related to mitochondrial proton and electron leaking that will have a very detrimental effect on coupling efficiency (Treberg et al., 2018).

## Discussion

The human body comprises about 40 trillion cells, each one containing up to 6 billion proteins—the entire system being in continual change to adapt to either internal or external changing conditions (Moseley, 2013; Noack and Wiechert, 2014). It is well acknowledged that slight cell modifications could easily impair protein expression or activity levels thus altering global system metabolic performances leading into diseases. Moreover, some drugs affect cell function in an acute way while others may lead to cell dysfunction in a continuous manner leading with time to chronic diseases. This is particularly true for mitochondrial diseases as slight mitochondrial alteration could lead to global cellular dysfunction. To overcome limitations of conventional explorations, combination of multiple technologies is mandatory. Data aggregation of combined omics technologies may represent an elegant way to solve the problem. However, in most studies, static quantities of one biomolecules class (DNA, RNA, proteins, metabolites) are measured but do not preclude on system functionality. Transcriptomics allows semi-quantitative determination of transcripts but does not give any information on their translatability. As post-transcriptional and post-translational modifications are highly regulated, no information regarding protein synthesis and activity could then be derived. It holds true for proteomics that allows quantification of large number of proteins but does not preclude on their functionality. Indeed, proteins displaying activity (such as enzymes) can be present in a relative amount, but no one knows whether it has been activated and able to work. Measuring metabolites using metabolomics allows visualizing metabolic chains with intermediate and/or end-products but does not give any information on functionality of cascade enzymes. To bypass such limitations, monitoring temporal changes of any molecules in the body is interesting. However, either newly synthetized products or clearance level of already existing ones need to be observed. For that purpose, fluxomics have been developed using stable isotopes in kinetics studies (Kohlstedt et al., 2010) allowing determination of metabolites intracellular flux (Claydon and Beynon, 2012; Previs and Kelley, 2015). Nevertheless, as it is very expensive and heavy to process, isotope use has been limited.

One alternative way to address a dynamic issue should be sampling at different times. However, even if measurement iterations are performed, it presumes that quantitative measurement techniques are sufficiently precise and reproducible to be able to compare results in a dynamic fashion. For quantification, overall variance of biological measurement can be deconvoluted into specific variances due to either nature of sample that would be studied or methods that would be used to perform quantification (Figure 1). Living material is notoriously variable and most variations are unpredictable. Thus, the experimenter has only little to no control on them. For example, it remains tricky to separate viable from non-viable cells in cell culture dish as it is also largely problematic to evidence exact percentage of tumor versus normal cells in a tissue, but efforts can be made to lower these errors which may impair further interpretation. Pre-analytical and analytical phases are also very significant. As an important source of variability that can be controlled (Nunnally et al., 2008), both must be correctly mastered. It is commonly admitted that processing any measurement with bad starting sample will undoubtedly generate awful results. Many studies have evidenced, more notably in the field of clinical biology as biological results have direct impact on patient’s handling, that most of errors are found outside the analytical phase. Moreover, pre-analytical steps have been found to be the most vulnerable to risk of error. To limit their impact, recent technical recommendations regarding sampling, storage, transport and identification have been developed by consensus organizations (Lippi et al., 2015).

In industrial health research, whereas relative quantification may lead to meaningful results during early stages of drug development, transfer to clinical biology must be considered as a major goal as it represents a validation of its usefulness. Clinical biology criteria in terms of accuracy and precision, are largely more stringent than research criteria. As high CV%s tests display low power to detect small-scale differences (Figure 2), low CV% tests are generally preferred as they can significantly detect very small variations. Generally, the only way to compensate and increase high CV% techniques relies in increasing replicates number thus increasing starting material. Using tests with the least inherent variability will induce the least replication and thus will be the less material consumer thus the most cost-effective. Indeed, due to high precision level, experiments can be performed only once for each sample like what is routinely done for biomarkers in patient’s blood samples. Such procedure saves sample volumes and allows carrying out a great number of experiments on the same plasma aliquot.

Using our new platform, we have primarily focused on mitochondrial function and oxidative stress that are commonly linked together. Indeed, catalytic dysfunctions in mitochondrial ETC and/or OS pathways that lead to bioenergetics defects have been reported in large number of species (from unicellular to multicellular eukaryotes, from vegetal to animal) (Schwarzländer and Finkemeier, 2013; Song et al., 2016). In mammals, whereas OS seems ubiquitously distributed, ETC dysfunction is frequently observed in specialized tissues. As compound evaluation can be performed either in preclinical or clinical stages, development of versatile diagnostic assays usable either on a large subset of cells and/or tissues or on a variety of living species (from yeast to humans) is strongly mandatory. Traditional quantification methods either for ETC or OS are usually labor-intensive and time-consuming because it is still largely manual. Thus, they are only available in a small number of very specialized laboratories able to extract with appropriate means mitochondria from whole cells. Most of conventional techniques lack analytical performances, especially precision, with intra- and inter-CV% largely above 10%, impairing detection of tiny variations that are very common in chronic illnesses. Traditionally, measurement of a large panel of markers requires the use of a large range of different techniques. It also requires measuring parameters successively and never in parallel, generating an unavoidable degradation of starting material.

The use of a random-access analyzer for automated analysis of mitochondrial enzymes is not new as it was originally described in cultured skin fibroblasts by Williams et al. (1998) and later extended to the four complexes of the respiratory chain (Kramer et al., 2005). Such automated method was faster to perform, less expensive, and required less than one half of the sample material needed for traditional manual methods. Moreover, as multiparametric analyzers are now able to quantify in parallel up to 40 different biomarkers, it becomes possible on a unique sample to uncover different metabolic parts of the cellular machinery.

When applied to cell lines, as cellular enzymes work together, it becomes possible to evaluate the relative importance of each metabolic cascade. At the light of our results, it is tempting to postulate that, when compared to PNT1A normal prostate cells, both VCaP and PC3 prostate cancer cells undergo OS but this OS is still well controlled (limited lipid peroxidation) by increased global antioxidant defense. This strong capability of CaP cells to efficiently fight OS is certainly a cardinal feature that allows them to resist to conventional prooxidative chemotherapy. It is also possible to evaluate the influence of a metabolite (like glucose in Figures 5A,B) or a drug on cell metabolism (like rotenone in Figures 6A,B). The use of reliable techniques allows comparison between cell lines and evidence that cells behave differently under treatment (Figures 5, 6). It is also interesting to notice that a well-known ETC complex I inhibitor can have effects not only on complex I but also on other parts of metabolism (it decreases ATP production but, for yet unknown reasons, it lowers also GPX activity) (Figure 6A). When applied *in vivo* to specific tissues or whole animal, (Figure 6B), it allows to understand the impact a compound may have on cell metabolism and how the cell will react to its presence. Such results are largely complementary to omics data. Identifying transcripts, metabolites or protein do not preclude whether the system is functional or not. The ability to measure enzyme activities gives invaluable information not only on protein amount but also on protein functionality. Looking at the results, the enzyme is present in a given amount but also is more or less active. Combining this enzyme activity with others in the same cellular metabolic pathway allows getting an insight on the overall functionality of this pathway. In addition, measuring, in parallel on the same starting material, different parameters allow the establishment of ratios (as enzyme activity ratios). Because CV% of each enzymatic technique is below 5%, it becomes possible to compare these ratios. As it is also possible to very precisely measure protein sample concentration in the same set of measurements, all the quantitative tests can be reported to this content and can be compared together. Using such ratios may have an importance to unravel complex metabolism features. As an example, looking at OS cellular effects creates the thought that an observed SOD increase may certainly have a cellular benefit as it reflects a positive cell response. Nevertheless, as superoxide anion detoxification leads to detrimental H_2_O_2_ production, a parallel increase in GPX activity is mandatory to reduce H_2_O_2_. Thus, a stable GPX/SOD ratio may reflect an efficient cellular response while a decreased one may indicate a decrease in H_2_O_2_ reducing power that will certainly have deleterious consequences such as cellular peroxidation of multiple cellular elements. This peroxidation can lead to read-outs modification such as increased GSH consumption and/or potential MDA increase. Indeed, the use of tests panel rather than a single test in such complex metabolic cascades greatly increases both analysis quality and interpretation fineness. Thus, performing in parallel several panels of tests uncovering multiple metabolic pathways will definitely pave the way to metabolic integration.

At the scale of a multi-person study on the effect of a compound, it becomes possible to perform enzyme measurements on tissues from a large set of individuals and easily detect outliers that behave differently (Figure 8). Because of the very low CV% techniques, aside comparison of drug effect on cell or organ tissue behavior, it can also be used to evaluate dose effects on whole animal (Figure 6B), or time-dependent drug effects on human cells (Figure 5B), measuring effects not only in a static but also in a dynamic fashion. In the latter case, if a time-dependent experiment can be designed, all samples are analyzed in parallel on the same apparatus at the same time with the same reagents and technical constraints, hereby limiting possible drawbacks (batch effect).

To increase results reliability and comparability between cell culture, animal testing or human follow-up, from preclinical studies to clinical ones, it seems essential to perform tests using the same technologies. Metabolites and enzyme activities measurements are strong biomarker candidates as they encompass species differences. Indeed, as shown in our example studies, enzyme activities behave globally similarly either in *Caenorhabditis* Elegans and rat tissues or in human white blood cells.

In the chemical toxicity testing field, new efficient and reliable methodologies are strongly required by regulatory agencies. By its versatility (from cultured cells and small animals to humans with the same analytical performances), its high precision and robustness, its high throughput capabilities (some analyzers may launch from 2,000 to 4,000 tests/h), our platform fulfill some of the NAMs requested features. As it may be expanded to different central cellular metabolisms (parallel implementation on the platform of other metabolisms, i.e., glycolysis, TCA cycle, fatty acids degradation, ketogenesis), it can give very precise information regarding the effect a compound may have on global cellular metabolism and bioenergetics. As an example, in humans, compound dose- or time-dependent effects can be observed either on circulating cells (white or red blood cells depending on the need of an OXPHOS activity evaluation) or specific tissue biopsies with the same accuracy.

## Conclusion

Assessing global functional metabolic behavior earlier and more comprehensively in drug development process will help avoid costly late-stage attrition, and more importantly, will improve drug safety. As more novel drugs, heading for restoring congruous cell, tissue or organ equilibrium in every disease that is metabolic in nature, are supposed to be developed in the future, the probability of concomitant mitochondrial and cytoplasmic metabolic pathways dysfunctions will largely increase. Because most of the defects are likely to be insidious, they will necessitate more sensitive, frequent and dynamic evaluation of complexes functionalities.

As complementary to classical “omics” studies, the use of multi-parametric equipment able to measure several markers in parallel with high precision, without any bias due to either inter-experiments variability or sample degradation, is certainly an interesting opportunity to evaluate compound impact along the different phases of its development. By its unique characteristics, its versatility (working from early pre-clinical to late clinical phases), its development capabilities, such platform will certainly help strategic decisions regarding subsequent drug testing or development.

## Data Availability

The raw data supporting the conclusion of this article will be made available by the authors, without undue reservation.

## References

[B1] Baker M. (2016). Reproducibility: Respect Your Cells!. Nature 537, 433–435. 10.1038/537433a 27629646

[B2] Barja G. (1999). Mitochondrial Oxygen Radical Generation and Leak: Sites of Production in States 4 and 3, Organ Specificity, and Relation to Aging and Longevity. J. Bioenerg. Biomembr 31, 347–366. 10.1023/a:1005427919188 10665525

[B3] Beutler E. Mitchell M. (1968). Brief Report: Special Modifications of the Fluorescent Screening Method for Glucose-6-Phosphate Dehydrogenase Deficiency. Blood 32, 816–818. 10.1182/blood.v32.5.816.816 4386875

[B4] Bhatti J. S. Bhatti G. K. Reddy P. H. (2017). Mitochondrial Dysfunction and Oxidative Stress in Metabolic Disorders - A Step towards Mitochondria Based Therapeutic Strategies. Biochim. Biophys. Acta (Bba) - Mol. Basis Dis. 1863, 1066–1077. 10.1016/j.bbadis.2016.11.010 PMC5423868 27836629

[B5] Blake R. Trounce I. A. (2014). Mitochondrial Dysfunction and Complications Associated with Diabetes. Biochim. Biophys. Acta (Bba) - Gen. Subjects 1840, 1404–1412. 10.1016/j.bbagen.2013.11.007 24246956

[B6] Bradford M. M. (1976). A Rapid and Sensitive Method for the Quantitation of Microgram Quantities of Protein Utilizing the Principle of Protein-Dye Binding. Anal. Biochem. 72, 248–254. 10.1016/0003-2697(76)90527-3 942051

[B7] Carbonell J. Marco R. Feliu J. E. Sols A. (1973). Pyruvate Kinase. Classes of Regulatory Isoenzymes in Mammalian Tissues. Eur. J. Biochem. 37, 148–156. 10.1111/j.1432-1033.1973.tb02969.x 4729424

[B8] Chance B. Sies H. Boveris A. (1979). Hydroperoxide Metabolism in Mammalian Organs. Physiol. Rev. 59, 527–605. 10.1152/physrev.1979.59.3.527 37532

[B9] Claydon A. J. Beynon R. (2012). Proteome Dynamics: Revisiting Turnover with a Global Perspective. Mol. Cell Proteomics 11, 1551–1565. 10.1074/mcp.o112.022186 23125033 PMC3518130

[B10] Conti M. Morand P. C. Levillain P. Lemonnier A. (1991). Improved Fluorometric Determination of Malonaldehyde. Clin. Chem. 37, 1273–1275. 10.1093/clinchem/37.7.1273 1855301

[B11] Dröge W. (2002). Free Radicals in the Physiological Control of Cell Function. Physiol. Rev. 82, 47–95. 10.1152/physrev.00018.2001 11773609

[B12] Ein-Dor L. Zuk O. Domany E. (2006). Thousands of Samples Are Needed to Generate a Robust Gene List for Predicting Outcome in Cancer. Proc. Natl. Acad. Sci. 103, 5923–5928. 10.1073/pnas.0601231103 16585533 PMC1458674

[B13] Endlicher R. Křiváková P. Rauchová H. Nůsková H. Červinková Z. Drahota Z. (2009). Peroxidative Damage of Mitochondrial Respiration Is Substrate-dependent. Physiol. Res. 58, 685–692. 10.33549/physiolres.931635 19093725

[B14] Finkel T. Holbrook N. J. (2000). Oxidants, Oxidative Stress and the Biology of Ageing. Nature 408, 239–247. 10.1038/35041687 11089981

[B15] Fondi M. Liò P. (2015). Multi -omics and Metabolic Modelling Pipelines: Challenges and Tools for Systems Microbiology. Microbiol. Res. 171, 52–64. 10.1016/j.micres.2015.01.003 25644953

[B16] Forbes J. M. Cooper M. E. (2013). Mechanisms of Diabetic Complications. Physiol. Rev. 93, 137–188. 10.1152/physrev.00045.2011 23303908

[B17] Ghosh D. Poisson L. M. (2009). "Omics" Data and Levels of Evidence for Biomarker Discovery. Genomics 93, 13–16. 10.1016/j.ygeno.2008.07.006 18723089

[B18] Go Y.-M. Fernandes J. Hu X. Uppal K. Jones D. P. (2018). Mitochondrial Network Responses in Oxidative Physiology and Disease. Free Radic. Biol. Med. 116, 31–40. 10.1016/j.freeradbiomed.2018.01.005 29317273 PMC5833979

[B19] Houstis N. Rosen E. D. Lander E. S. (2006). Reactive Oxygen Species Have a Causal Role in Multiple Forms of Insulin Resistance. Nature 440, 944–948. 10.1038/nature04634 16612386

[B20] Huang C. S. Chang L. S. Anderson M. E. Meister A. (1993). Catalytic and Regulatory Properties of the Heavy Subunit of Rat Kidney Gamma-Glutamylcysteine Synthetase. J. Biol. Chem. 268, 19675–19680. 10.1016/s0021-9258(19)36569-x 8103521

[B21] Huet O. Dupic L. Batteux F. Matar C. Conti M. Chereau C. (2011). Postresuscitation Syndrome: Potential Role of Hydroxyl Radical-Induced Endothelial Cell Damage*. Crit. Care Med. 39, 1712–1720. 10.1097/ccm.0b013e3182186d42 21494109

[B22] Hüttemann M. Lee I. Samavati L. Yu H. Doan J. W. (2007). Regulation of Mitochondrial Oxidative Phosphorylation through Cell Signaling. Biochim. Biophys. Acta (Bba) - Mol. Cel Res. 1773, 1701–1720. 10.1016/j.bbamcr.2007.10.001 18240421

[B23] Ide T. Tsutsui H. Kinugawa S. Utsumi H. Kang D. Hattori N. (1999). Mitochondrial Electron Transport Complex I Is a Potential Source of Oxygen Free Radicals in the Failing Myocardium. Circ. Res. 85, 357–363. 10.1161/01.res.85.4.357 10455064

[B24] Ioannidis J. P. A. (2010). Expectations, Validity, and Reality in Omics. J. Clin. Epidemiol. 63, 945–949. 10.1016/j.jclinepi.2010.04.002 20573481

[B25] Iommarini L. Ghelli A. Gasparre G. Porcelli A. M. (2017). Mitochondrial Metabolism and Energy Sensing in Tumor Progression. Biochim. Biophys. Acta (Bba) - Bioenerg. 1858, 582–590. 10.1016/j.bbabio.2017.02.006 28213331

[B26] Iskar M. Zeller G. Zhao X.-M. van Noort V. Bork P. (2012). Drug Discovery in the Age of Systems Biology: the Rise of Computational Approaches for Data Integration. Curr. Opin. Biotechnol. 23, 609–616. 10.1016/j.copbio.2011.11.010 22153034

[B27] Itoh H. Srere P. A. (1970). A New Assay for Glutamate-Oxaloacetate Transaminase. Anal. Biochem. 35, 405–410. 10.1016/0003-2697(70)90202-2 5450436

[B28] Itoh K. Nakamura K. Iijima M. Sesaki H. (2013). Mitochondrial Dynamics in Neurodegeneration. Trends Cel Biol. 23, 64–71. 10.1016/j.tcb.2012.10.006 PMC3558617 23159640

[B29] James A. M. Murphy M. P. (2002). How Mitochondrial Damage Affects Cell Function. J. Biomed. Sci. 9, 475–487. 10.1007/bf02254975 12372986

[B30] Johansson L. H. Håkan Borg L. A. (1988). A Spectrophotometric Method for Determination of Catalase Activity in Small Tissue Samples. Anal. Biochem. 174, 331–336. 10.1016/0003-2697(88)90554-4 3064653

[B31] Kamarajugadda S. Stemboroski L. Cai Q. Simpson N. E. Nayak S. Tan M. (2012). Glucose Oxidation Modulates Anoikis and Tumor Metastasis. Mol. Cel Biol 32, 1893–1907. 10.1128/mcb.06248-11 PMC3347404 22431524

[B32] Karczewski K. J. Snyder M. P. (2018). Integrative Omics for Health and Disease. Nat. Rev. Genet. 19, 299–310. 10.1038/nrg.2018.4 29479082 PMC5990367

[B33] Klepinin A. Zhang S. Klepinina L. Rebane-Klemm E. Terzic A. Kaambre T. (2020). Adenylate Kinase and Metabolic Signaling in Cancer Cells. Front. Oncol. 10, 660. 10.3389/fonc.2020.00660 32509571 PMC7248387

[B34] Kohlstedt M. Becker J. Wittmann C. (2010). Metabolic Fluxes and Beyond-Systems Biology Understanding and Engineering of Microbial Metabolism. Appl. Microbiol. Biotechnol. 88, 1065–1075. 10.1007/s00253-010-2854-2 20821203

[B35] Krähenbühl S. Schäfer T. Wiesmann U. (1996). Determination of the Activities of the Enzyme Complexes of the Electron Transport Chain in Human Fibroblasts. Clinica Chim. Acta 253, 79–90. 10.1016/0009-8981(96)06338-3 8879840

[B36] Krähenbühl S. Talos C. Wiesmann U. Hoppel C. L. (1994). Development and Evaluation of a Spectrophotometric Assay for Complex III in Isolated Mitochondria, Tissues and Fibroblasts from Rats and Humans. Clinica Chim. Acta 230, 177–187. 10.1016/0009-8981(94)90270-4 7834868

[B37] Kramer K. A. Oglesbee D. Hartman S. J. Huey J. Anderson B. Magera M. J. (2005). Automated Spectrophotometric Analysis of Mitochondrial Respiratory Chain Complex Enzyme Activities in Cultured Skin Fibroblasts. Clin. Chem. 51, 2110–2116. 10.1373/clinchem.2005.050146 16141288

[B38] Kramer P. Nowak T. (1988). The Preparation and Characterization of Cr(III) and Co(III) Complexes of GDP and GTP and Their Interactions with Avian Phosphoenolpyruvate Carboxykinas. J. Inorg. Biochem. 32, 135–151. 10.1016/0162-0134(88)80022-9 3346664

[B39] Lay J. O. Jr. Liyanage R. Borgmann S. Wilkins C. L. (2006). Problems with the “Omics”. Trac Trends Anal. Chem. 25, 1046–1056. 10.1016/j.trac.2006.10.007

[B40] Lay J. O. Jr. Liyanage R. Durham B. Brooks J. (2006). Rapid Characterization of Edible Oils by Direct Matrix-Assisted Laser Desorption/ionization Time-Of-Flight Mass Spectrometry Analysis Using Triacylglycerols. Rapid Commun. Mass. Spectrom. 20, 952–958. 10.1002/rcm.2394 16470678

[B41] Le Chen L. Knowlton A. A. (2011). Mitochondrial Dynamics in Heart Failure. Congest. Heart Fail. 17, 257–261. 10.1111/j.1751-7133.2011.00255.x 22848903 PMC3410538

[B42] Leung E. L. Cao Z.-W. Jiang Z.-H. Zhou H. Liu L. (2013). Network-based Drug Discovery by Integrating Systems Biology and Computational Technologies. Brief. Bioinform. 14, 491–505. 10.1093/bib/bbs043 22877768 PMC3713711

[B43] Liemburg-Apers D. C. Willems P. H. G. M. Koopman W. J. H. Grefte S. (2015). Interactions between Mitochondrial Reactive Oxygen Species and Cellular Glucose Metabolism. Arch. Toxicol. 89, 1209–1226. 10.1007/s00204-015-1520-y 26047665 PMC4508370

[B44] Lippi G. Banfi G. Church S. Cornes M. De Carli G. Grankvist K. (2015). Preanalytical Quality Improvement. In Pursuit of harmony, on Behalf of European Federation for Clinical Chemistry and Laboratory Medicine (EFLM) Working Group for Preanalytical Phase (WG-PRE). Clin. Chem. Lab. Med. 53, 357–370. 10.1515/cclm-2014-1051 25490032

[B45] Madamanchi N. R. Runge M. S. (2007). Mitochondrial Dysfunction in Atherosclerosis. Circ. Res. 100, 460–473. 10.1161/01.res.0000258450.44413.96 17332437

[B46] Markuszewski M. J. Britz-McKibbin P. Terabe S. Matsuda K. Nishioka T. (2003). Determination of Pyridine and Adenine Nucleotide Metabolites in Bacillus Subtilis Cell Extract by Sweeping Borate Complexation Capillary Electrophoresis. J. Chromatogr. A. 989, 293–301. 10.1016/s0021-9673(03)00031-1 12650262

[B47] McCord J. M. Fridovich I. (1969). Superoxide Dismutase. J. Biol. Chem. 244, 6049–6055. 10.1016/s0021-9258(18)63504-5 5389100

[B48] Misrani A. Tabassum S. Yang L. (2021). Mitochondrial Dysfunction and Oxidative Stress in Alzheimer's Disease. Front. Aging Neurosci. 13, 617588. 10.3389/fnagi.2021.617588 33679375 PMC7930231

[B49] Moseley H. N. (2013). Error Analysis and Propagation in Metabolomics Data Analysis. Comput. Struct. Biotechnol. J. 4 (5), e201301006. 10.5936/csbj.201301006 23667718 PMC3647477

[B50] Murphy M. P. (2009). How Mitochondria Produce Reactive Oxygen Species. Biochem. J. 417, 1–13. 10.1042/bj20081386 19061483 PMC2605959

[B51] Muscari C. Pappagallo M. Ferrari D. Giordano E. Capanni C. Caldarera C. M. (1998). Simultaneous Detection of Reduced and Oxidized Glutathione in Tissues and Mitochondria by Capillary Electrophoresis. J. Chromatogr. B: Biomed. Sci. Appl. 707, 301–307. 10.1016/s0378-4347(97)00595-1 9613963

[B52] Nadanaciva S. Will Y. (2011). Investigating Mitochondrial Dysfunction to Increase Drug Safety in the Pharmaceutical Industry. Cdt 12, 774–782. 10.2174/138945011795528985 21275886

[B53] Nadanaciva S. Will Y. (2011). New Insights in Drug-Induced Mitochondrial Toxicity. Cpd 17, 2100–2112. 10.2174/138161211796904795 21718246

[B54] Nass R. Hamza I. (2007). The Nematode *C. elegans* as an Animal Model to Explore Toxicology *In Vivo*: Solid and Axenic Growth Culture Conditions and Compound Exposure Parameters. Curr. Protoc. Toxicol. 31, 1–18. 10.1002/0471140856.tx0109s31 20922756

[B55] Noack S. Wiechert W. (2014). Quantitative Metabolomics: a Phantom. Trends Biotechnol. 32, 238–244. 10.1016/j.tibtech.2014.03.006 24708998

[B56] Nunnally B. K. Johnson A. Kaiser R. (2008). Reduction of Analytical Method Variability in a R&D Laboratory : Case Study. J. Valid Tech. 14, 77–82.

[B57] Ong S.-B. Hall A. R. Hausenloy D. J. (2013). Mitochondrial Dynamics in Cardiovascular Health and Disease. Antioxid. Redox Signaling 19, 400–414. 10.1089/ars.2012.4777 PMC3699895 22793879

[B58] Paglia D. E. Valentine W. N. (1967). Studies on the Quantitative and Qualitative Characterization of Erythrocyte Glutathione Peroxidase. J. Lab. Clin. Med. 70, 158–169. 6066618

[B59] Parish R. Petersen K. F. (2005). Mitochondrial Dysfunction and Type 2 Diabetes. Curr. Diab Rep. 5, 177–183. 10.1007/s11892-005-0006-3 15929863 PMC2995500

[B60] Perng W. Aslibekyan S. (2020). Find the Needle in the Haystack, Then Find it Again: Replication and Validation in the 'Omics Era. Metabolites 10 (7), 286, 1–13. 10.3390/metabo10070286 PMC7408356 32664690

[B61] Pesce F. Pathan S. Schena F. P. (2013). From -omics to Personalized Medicine in Nephrology: Integration Is the Key. Nephrol. Dial. Transplant. 28, 24–28. 10.1093/ndt/gfs483 23229923

[B62] Previs S. F. Kelley D. E. (2015). Tracer-based Assessments of Hepatic Anaplerotic and TCA Cycle Flux: Practicality, Stoichiometry, and Hidden Assumptions. Am. J. Physiology-Endocrinology Metab. 309, E727–E735. 10.1152/ajpendo.00216.2015 26330343

[B63] Rahman J. Rahman S. (2018). Mitochondrial Medicine in the Omics Era. The Lancet 391, 2560–2574. 10.1016/s0140-6736(18)30727-x 29903433

[B64] Reddy P. H. (2006). Mitochondrial Oxidative Damage in Aging and Alzheimer's Disease: Implications for Mitochondrially Targeted Antioxidant Therapeutics. J. Biomed. Biotechnol. 2006, 31372. 10.1155/JBB/2006/31372 17047303 PMC1559913

[B65] Reddy P. H. (2008). Mitochondrial Medicine for Aging and Neurodegenerative Diseases. Neuromol Med. 10, 291–315. 10.1007/s12017-008-8044-z PMC3235551 18566920

[B66] Richter C. Park J. W. Ames B. N. (1988). Normal Oxidative Damage to Mitochondrial and Nuclear DNA Is Extensive. Proc. Natl. Acad. Sci. 85, 6465–6467. 10.1073/pnas.85.17.6465 3413108 PMC281993

[B67] Rocha M. Rovira-Llopis S. Banuls C. Bellod L. Falcon R. Castello R. (2013). Mitochondrial Dysfunction and Oxidative Stress in Insulin Resistance. Cpd 19, 5730–5741. 10.2174/13816128113199990373 23448492

[B68] Rustin P. Lebidois J. Chretien D. Bourgeron T. Piechaud J.-F. Rötig A. (1993). The Investigation of Respiratory Chain Disorders in Heart Using Endomyocardial Biopsies. J. Inherit. Metab. Dis. 16, 541–544. 10.1007/bf00711676 7609447

[B69] Schumann G. Bonora R. Ceriotti F. Clerc-Renaud P. Ferrero C. A. Férard G. (2002). IFCC Primary Reference Procedures for the Measurement of Catalytic Activity Concentrations of Enzymes at 37 Degrees C. Part 3. Reference Procedure for the Measurement of Catalytic Concentration of Lactate Dehydrogenase. Clin. Chem. Lab. Med. 40, 643–648. 10.1515/CCLM.2002.111 12211663

[B70] Schwarzländer M. Finkemeier I. (2013). Mitochondrial Energy and Redox Signaling in Plants. Antioxid. Redox Signaling 18, 2122–2144. 10.1089/ars.2012.5104 PMC3698670 23234467

[B71] Serru V. Baudin B. Ziegler F. David J.-P. Cals M.-J. Vaubourdolle M. (2001). Quantification of Reduced and Oxidized Glutathione in Whole Blood Samples by Capillary Electrophoresis. Clin. Chem. 47, 1321–1324. 10.1093/clinchem/47.7.1321 11427471

[B72] Sherratt H. S. (1991). Efficacy and Tolerability of Calcipotriol in Psoriasis. Rev. Neurol. (Paris) 147, 417–423. 10.1515/9783110850345-130 1962047

[B73] Short K. R. Bigelow M. L. Kahl J. Singh R. Coenen-Schimke J. Raghavakaimal S. (2005). Decline in Skeletal Muscle Mitochondrial Function with Aging in Humans. Proc. Natl. Acad. Sci. 102, 5618–5623. 10.1073/pnas.0501559102 15800038 PMC556267

[B74] Snedecor G. W. Cochran W. G. (1967). Statistical Methods. Ames: Iowa State University Press.

[B75] Song G. Napoli E. Wong S. Hagerman R. Liu S. Tassone F. (2016). Altered Redox Mitochondrial Biology in the Neurodegenerative Disorder Fragile X-Tremor/ataxia Syndrome: Use of Antioxidants in Precision Medicine. Mol. Med. 22, 548–559. 10.2119/molmed.2016.00122 27385396 PMC5082295

[B76] Strunz S. Wolkenhauer O. de la Fuente A. (2016). Network-Assisted Disease Classification and Biomarker Discovery. Methods Mol. Biol. 1386, 353–374. 10.1007/978-1-4939-3283-2_16 26677191

[B77] Sung J. Wang Y. Chandrasekaran S. Witten D. M. Price N. D. (2012). Molecular Signatures from Omics Data: from Chaos to Consensus. Biotechnol. J. 7, 946–957. 10.1002/biot.201100305 22528809 PMC3418428

[B78] Treberg J. R. Munro D. Jastroch M. Quijada-Rodriguez A. R. Kutschke M. Wiens L. (2018). Comparing Electron Leak in Vertebrate Muscle Mitochondria. Integr. Comp. Biol. 58, 495–505. 10.1093/icb/icy095 30010782

[B79] Trinder P. (1969). Determination of Blood Glucose Using an Oxidase-Peroxidase System with a Non-carcinogenic Chromogen. J. Clin. Pathol. 22, 158–161. 10.1136/jcp.22.2.158 5776547 PMC474026

[B80] Uhrová M. Deyl Z. Suchánek M. (1996). Separation of Common Nucleotides, Mono-, Di- and Triphosphates, by Capillary Electrophoresis. J. Chromatogr. B: Biomed. Sci. Appl. 681, 99–105. 10.1016/0378-4347(95)00535-8 8798918

[B81] Van Assche R. Broeckx V. Boonen K. Maes E. De Haes W. Schoofs L. (2015). Integrating -Omics: Systems Biology as Explored through *C. elegans* Research. J. Mol. Biol. 427, 3441–3451. 10.1016/j.jmb.2015.03.015 25839106

[B82] Vigelsø A. Andersen N. B. Dela F. (2014). The Relationship between Skeletal Muscle Mitochondrial Citrate Synthase Activity and Whole Body Oxygen Uptake Adaptations in Response to Exercise Training. Int. J. Physiol. Pathophysiol Pharmacol. 6, 84–101. 25057335 PMC4106645

[B83] Williams A. J. Coakley J. Christodoulou J. (1998). Automated Analysis of Mitochondrial Enzymes in Cultured Skin Fibroblasts. Anal. Biochem. 259, 176–180. 10.1006/abio.1998.2624 9618194

[B84] Yin C. Qie S. Sang N. (2012). Carbon Source Metabolism and its Regulation in Cancer Cells. Crit. Rev. Eukar Gene Expr. 22, 17–35. 10.1615/critreveukargeneexpr.v22.i1.20 PMC4505802 22339657

[B85] Zapalska-Sozoniuk M. Chrobak L. Kowalczyk K. Kankofer M. (2019). Is it Useful to Use Several "omics" for Obtaining Valuable Results. Mol. Biol. Rep. 46, 3597–3606. 10.1007/s11033-019-04793-9 30989558

